# Dynamics of collective performance in collaboration networks

**DOI:** 10.1371/journal.pone.0204547

**Published:** 2018-10-10

**Authors:** Victor Amelkin, Omid Askarisichani, Young Ji Kim, Thomas W. Malone, Ambuj K. Singh

**Affiliations:** 1 Warren Center for Network & Data Sciences, Department of Electrical and Systems Engineering, University of Pennsylvania, Philadelphia, PA, United States of America; 2 Department of Computer Science, University of California Santa Barbara, Santa Barbara, CA, United States of America; 3 Department of Communication, University of California Santa Barbara, Santa Barbara, CA, United States of America; 4 MIT Sloan School of Management, MIT Center for Collective Intelligence, Massachusetts Institute of Technology, Cambridge, MA, United States of America; IBM Thomas J Watson Research Center, UNITED STATES

## Abstract

Today, many complex tasks are assigned to teams, rather than individuals. One reason for teaming up is expansion of the skill coverage of each individual to the joint team skill set. However, numerous empirical studies of human groups suggest that the performance of equally skilled teams can widely differ. Two natural question arise: *What are the factors defining team performance?* and *How can we best predict the performance of a given team on a specific task?* While the team members’ task-related capabilities constrain the potential for the team’s success, the key to understanding team performance is in the analysis of the *team process*, encompassing the behaviors of the team members during task completion. In this study, we extend the existing body of research on team process and prediction models of team performance. Specifically, we analyze the dynamics of historical team performance over a series of tasks as well as the fine-grained patterns of collaboration between team members, and formally connect these dynamics to the team performance in the predictive models. Our major qualitative finding is that *higher performing teams have well-connected collaboration networks*—as indicated by the topological and spectral properties of the latter—which are more robust to perturbations, and where network processes spread more efficiently. Our major quantitative finding is that *our predictive models deliver accurate team performance predictions*—with a prediction error of 15-25%—on a variety of simple tasks, outperforming baseline models that do not capture the micro-level dynamics of team member behaviors. We also show how to use our models in an application, for optimal online planning of workload distribution in an organization. Our findings emphasize the importance of studying the dynamics of team collaboration as the major driver of high performance in teams.

## 1 Introduction

Teams are now a basic unit of knowledge work. Organizations increasingly rely on teams, as work has become complex enough to require a wide variety of skills and expertise from a group of individuals [1]. Scientific knowledge is increasingly produced by teams of researchers instead of an individual author [2]. Research shows that teams produce better outcomes than individuals alone for complex knowledge work. For example, science research by teams has been more impactful and novel than solo work [3, 4]. Given the importance of teams in knowledge work, the two natural questions are *What are the factors that contribute to team performance?* and *How can we exploit these factors to make accurate predictions of team performance?* Our study is dedicated to answering these two questions.

Team performance is commonly considered an output of the input-process-output (I-P-O) model [5], the widely known conceptual framework for studying groups. Research based on the I-P-O model tends to assume that inputs such as group composition, lead to processes, which in turn lead to outcomes such as performance [6]. One apparent input factor of team performance is the task-related proficiency of the team members, and has been studied in various disciplines. In computer science and engineering, a large number of studies focus on optimal team design, with team optimality typically being defined in terms of the skill coverage of team members [7–9] or team members’ skill diversity [10]. Similarly, much of social scientific research focuses on the impact of cognitive abilities of team members upon the team’s performance (see the reviews of Kozlowski and Ilgen [11] and Stewart [12]). However, the team members’ proficiencies define the *potential* for good team performance, constraining rather than defining the actual performance. The empirical studies as early as the 1949 work of Deutsch [13] have shown that collaboration and cooperation are important contributors to team performance. More recently, Barron has shown in her study [14] of the performance of small teams of students on solving mathematical problems that equally competent teams can perform very differently depending on *how these teams’ members work together*. Similarly, Devine and Philips [15] have shown the lack of connection between the variance of team members’ cognitive abilities and the team performance; an analogous result has been reported by Shim and Srivastava [16] in their study of massively multiplayer online role-playing games. The discrepancy between team members’ individual abilities and the team’s performance is attributed to team processes, which mediate the translation of inputs to outputs [11]. The critical dependence of team performance outcomes upon the team process is also assumed in existing works on transactive memory systems [17], where team performance depends on team members’ efficiently learning each other’s capabilities, and has also been recently studied by Grand et al. [18] in the context of tasks the success on which heavily depends on efficient knowledge sharing. Thus, in studying team performance, it is essential to investigate the *team process*, defined as the actions and interactions team members engage in while working on tasks.

While team processes are dynamic in nature, the ways in which they are studied have been rather static [11]. In both social sciences and engineering, the team process has been extensively studied indirectly—without explicitly measuring cross-member interaction—through the analysis of the team members’ *potential for efficient collaboration*. In computer science literature, the problem of team formation in the presence of social network has been studied from an algorithmic perspective [8, 19–21]. The basic assumption in these works is that, in order to succeed in the completion of a task, not only the team members’ aggregate proficiencies should be sufficient, but the team members should also be able to efficiently communicate by being “mutually compatible” as manifested by their proximity in the social network they are embedded in. In social sciences, works that claim to study team process have often selected variables that do not represent actual interaction processes [22]. In response to the common tendency that team processes are often measured by summarizing individual team members’ attitudes, values, cognitions, and motivations at the team level, researchers call for defining these constructs as emergent states while distinguishing them from team processes, which directly denote team member interaction processes [22].

Recently, team process has been studied directly, through the explicit analysis of some traits of team members’ communication. For example, Brewer et al. [23] empirically study how miscommunication affects team performance as well as the ways to alleviate this effect. Goodman et al. [24] study how the communication delays in human-machine teams affect the latter’s performance. Kamrani et al. [25] propose a theoretical predictive model of team performance incorporating interaction between team members and its effect on the team’s performance. Similarly, Jiang et al. [26] develop a team performance model capturing congruence between the actual amount of cross-member communication and the amount of communication required by the task at hand. Jung [27] studies the connection between the dynamics of intra-team conflict or “the mood” of communication and the team performance. Finally, Barron [14] has empirically demonstrated that the performance of equally skilled teams depends on how these teams’ members react to each others’ task solution proposals.

Based on the above review of existing literature, it is clear that understanding team performance requires looking beyond team composition based on team members’ individual characteristics. Furthermore, high-level team process characteristics studied in the previous literature—such as the number of turns taken in conversation, or proximity of team members in a social network—are insufficient because of their “static” nature as well as being overly coarse characteristics of the cross-member interaction. Thus, in order to gain a deeper insight into the nature of team performance and design predictive models allowing its accurate prediction, we need to look at the dynamics of the team process as well as the fine-grain patterns of team members’ interaction.

The central goal of this study is to design formal predictive models for team performance that would use the knowledge of the intra-team process’ dynamics. Particularly, we focus on two types of dynamics: the dynamics of historical team performance, as well as the dynamics of the interaction among team members within their collaboration networks.

The data we analyze was collected by Engel et al. [28] to study the performance of small human groups on simple task sequences. In addition to the performance data, which was the primary focus of the original study, we also do extensive analyses of the team member communication logs that Engel et al. collected but did not extensively analyze.

We make the following *three specific contributions*.

1. Our analysis of the connection of team performance with the dynamics of historical team performance reveals that most teams with high average performance start performing well early and perform consistently well throughout the entire task battery. Lower-performing teams, on the other hand, start poorly, with their performance’s noticeably and steadily improving over time.
2. Our analysis of the interaction between team members reveals that the high-performing teams have well-connected collaboration networks, characterized by high mean and low variance of node degree, high mean edge density and reciprocity, and high algebraic connectivity. Such networks are known to be robust, and allowing for an efficient spread of network processes, such as consensus seeking.
3. Our predictive models of team performance allow for accurate performance predictions—with a relative error of 15-25%—on a variety of simple tasks, outperforming baseline models that do not capture the fine details of the team process. Additionally, we show how our model based on the team performance dynamics history augmented with an outlier control mechanism can be applied to the problem of optimal online planning of workload distribution in an organization. When used in this way, our model outperforms the baselines.

Our work reinforces the idea that the team performance is largely determined by *how* the team members collaborate, and emphasizes the importance of studying the dynamics of team collaboration as a major driver of high performance in teams.

The remainder of our paper is organized as follows. In Sec. 2, we describe the experimental setting as well as the collected team features characterizing the teams’ composition, performance, and communication. In Sec. 3, we describe our general team performance modeling approach (Sec. 3.1) as well as the specific models—the model based on the dynamics of historical team performance (Sec. 3.2) and the model based on the collaboration patterns (Sec. 3.3). Subsequently, in Sec. 4.1, 4.2, and 4.3, respectively, we report the team performance modeling results. In the subsequent Sec. 5, we show how to use one of our models for optimal workload distribution planning and compare our model’s performance to that of the baselines. Finally, in Sec. 6, we conclude with the discussion of our results as well as directions for future research. Additional description of the data, as well as the data itself are provided in S1 Dataset.

## 2 Data

In this work, we analyze the data of Engel et al. [28], which was collected to examine a group’s collective intelligence, defined as a group’s ability to perform a wide range of tasks [29]. In the study, 272 individuals were recruited from the general population of the Boston area via the Internet advertisements and divided into 68 four-member gender-balanced groups—with the average share of women across all the teams being around 49%—and asked to work on a series of group tasks for approximately an hour. The tasks were administered on a browser-based platform, which supports synchronous group collaboration as well as text-based chat. Each member used a laptop computer to work on the group tasks while communicating with other members. About a half of the groups were allowed to talk face-to-face while working on the tasks, while the other groups communicated via text chat only.

### 2.1 Tasks and team performance

All groups completed a battery of diverse group *tasks*, which represent five broad task categories described below (the tasks are executed by each group in the same order as they are listed here). The descriptions of all the tasks are provided in S1 Text.

1. Executing (*Typing Text*, *Typing Numbers*)
2. Sensing (*Detection Words*, *Detection Images*)
3. Generating (*Brainstorm Words*, *Brainstorm Brick*, *Brainstorm Equations*)
4. Choosing (*Matrix Solving*, *Unscramble Words*, *Sudoku*, *Judgement Slogans*, *Judgement Pictures*, *Judgement Pages*)
5. Memorizing (*Memory Video*, *Memory Images*)

**Fig 1:**
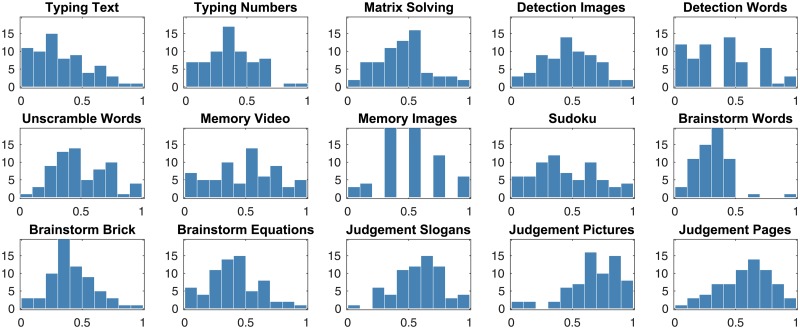
The distributions of task scores for 15 tasks for 68 groups.

For the completion of each task, each group receives a *task score*—reflecting the quality of the task completion result—on a task-specific scale. In our analysis, however, we linearly transform all the task scores to fit the range between 0.0 and 1.0; the corresponding task score distributions for all tasks are shown in Fig 1. We also used z-score normalization for the task scores as an alternative, but the simpler linear transform reported here resulted in more accurate predictive models for team performance.

### 2.2 Baseline team features

The data also includes the following *team features*—that we will use to establish a *baseline* performance prediction method—characterizing each team’s composition and summarizing its communication behavior:

⊳ proportion of female team members;
⊳ Big-5 personality traits [30] (e.g., average extraversion);
⊳ average social perceptiveness as measured by the “Reading the Mind in the Eyes” test [31], and
⊳ basic group communication features: the total amount of communication (the amount of time speaking for the face-to-face condition or the number of words typed for the online condition), and the distribution of communication (the standard deviation of these two measures).

**Fig 2:**
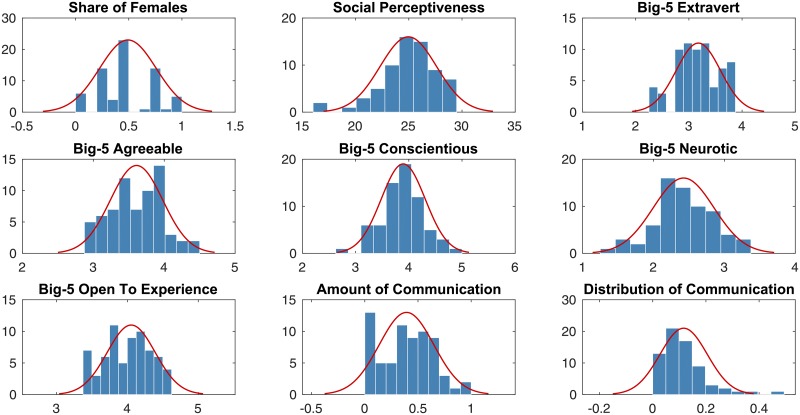
The value distributions of baseline team features for 68 teams.

The value distribution for each of the baseline features is shown in Fig 2.

## 3 Method

In what follows, we will, first, describe in Sec. 3.1 our general method for team performance analysis, and derive in Sec. 3.2 and 3.3, respectively, two qualitatively different sets of team features to be used with the general method.

### 3.1 General method

Our team feature-based analysis of team performance will consist of two stages.

At the first stage, we will study Pearson correlation between the team features as well as between team features and the scores that teams have earned for the completion of different tasks. The obtained p-values will be corrected using Benjamini-Hochberg procedure [32] with the false discovery rate of 10%. Further, only statistically significant correlation values will be reported, with the p-value threshold’s being 0.05. This analysis will provide an insight into which team features are “redundant” and which are statistically related to team performance.

The second stage of our analysis will be using team features to build a predictive model for team performance on each task. To that end, we will use elastic net regression [33], whose loss function, besides having the least-squares fitness term, is augmented with the LASSO [34] and ridge [35] regularizing terms:

$$\hat{\beta }=\underset{\beta }{\text{arg}\;\text{min}}\underset{\begin{array}{c} \text{least}\\ \text{squares} \end{array}}{(\underset{︸}{∥y-X\beta {∥}_{2}^{2}}}+\underset{\text{LASSO}}{\underset{︸}{\lambda_{1}∥\beta {∥}_{1}}}+\underset{\text{ridge}}{\underset{︸}{\lambda_{2}∥\beta {∥}_{2}^{2}}),}$$ (1)

where *β* is a model; ***y*** is a (response) vector of scores for a given task; ***X*** is the design matrix with its rows corresponding to teams, its columns corresponding to team features standardized to have zero mean and unit variance, and its entries being the team feature values; and *λ*_1_ and *λ*_2_ are the constant regularization parameters. The *ℓ*_1_-regularization term sparsifies the model trying to emphasize only a small number of significant team features, while the *ℓ*_2_-regularization term attempts to group related team features together, forcing the coefficients of highly-correlated features to have similar coefficients in model *β*. To deal with overfitting, we perform 10-fold cross-validation, considering different combinations of *λ*_1_ and *λ*_2_ that result in multiple regression models. Among these models, we select only those sparse enough, having the total weight of the top 6 features in the model accounting for at least 80% of the total model’s weight. Then, from these models, we choose the single best-fitting model, having the lowest root mean square error (RMSE). For readability, in the introduction of this manuscript, the prediction errors are reported in percentages, with an error of, say, 25% corresponding the RMSE of 0.25 for a prediction of a task score measured on a scale between 0.0 and 1.0.

In addition to regularized linear regression, we have also experimented with non-linear regression, Gaussian Process [36], as well as Support Vector Regression [37], yet, the best results were obtained using the simplest regularized linear regression, so we do not report the results for non-linear models.

Having found the best regression model for each task, we will analyze which team features are most emphasized in these models and, consequently, are predictive of team performance. Then, we will quantify the predictive quality of the obtained models, by making the actual team performance predictions and measuring the prediction accuracies.

### 3.2 Team performance dynamics-based features

The baseline team features described in Sec. 2 characterize the teams’ composition and summarize their basic behavioral properties, not capturing the temporal component of team work. In this section, we would like to understand whether the teams’ performance on early tasks is predictive of their future performance. To that end, for each team, and for each task the performance on which is to be predicted, we analyze the series of the task scores for the tasks the team has already completed, and extract from each such series the following features characterizing the historical dynamics of team performance:

⊳ the scores for the first and the last tasks;
⊳ the mean and the median scores;
⊳ the scores’ standard deviation and variance;
⊳ the scores’ skewness and kurtosis;
⊳ the features of the best line fitting the series (least squares fitting):
  – the ordinate values corresponding to the first, middle, and last tasks;
  – the slope, in radians;
  – the difference between the last and the first score;
⊳ the number of increases and decreases in a task score series; and
⊳ the number of score changes above and below the median score change value.

### 3.3 Collaboration pattern-based features—Teams as networks

The team features described so far are insufficient for our study. The baseline team features of Sec. 2 provide useful information about each team’s composition, yet, do not capture the fine details of the team members’ behavior. The basic communication features, such as the number of turns taken in conversation or the number of words spoken, do provide a limited information about the teams’ behavior, yet, do not capture how individual team members contribute to the team’s common goal and interact with their teammates.

In this section, we would like to understand how the intra-team interaction during task completion affects the quality of the outcome. In order to analyze the connection of the team members’ behavior patterns with their teams’ performance, we represent each team as a collaboration network, constructed as follows.

As mentioned in Sec. 2, about 50% of the participating teams were communicating only via an online text chat and were not allowed to talk to each other, despite residing in the same room while working on tasks. The communication data for these non-talking teams was recorded, with an example of a partial text chat log of a single team shown in Table 1.

**Table 1:** An example of a partial text chat log recorded while a non-talking team was working on tasks.

Time	Sender	Message
36:38	nick	So, I think the salt is really low importance.
37:00	kate	i don’t think the aircraft compass or motor oil are very useful
37:05	nick	kate, sun umbrella is high (we need shade!)
37:11	kate	i would put umbrella and food next after water
37:16	nick	Well, I think the compass works regardless, but yeah.
⋮	⋮	⋮
38:13	jeral	Mirror is high because it’s a signal
38:17	jeral	You can reflect the sun
38:25	greg	depends how big it is
38:29	kate	well yeah

Each message is accompanied by its timestamp and the sender’s alias.

Our immediate goal in the analysis of each such chat log is to extract the network that would describe *how the members of the team collaborate* in the process of task completion. While that information can, potentially, be derived from the text messages occurring in the logs, an automatic extraction of such collaboration semantics from the actual words included in short text messages is very challenging. Due to that fact, we will perform the analysis of team members’ collaboration solely based on the chat messages’ timestamps and senders. This approach implies a basic assumption: *if a message B appears on a chat log close enough in time to an earlier sent message A, then B is likely a response to A; and, the larger the time gap between two messages is, the less likely the later message is a response to the earlier message*. Following this assumption, for a message *m* occurring at time *m*.*time* on the log, we define the set of its responses

$$\begin{array}{c} R\left(m\right)=\left\{r|m.time<r.time\wedge t_{1}\leq r.time-m.time\leq t_{2}\wedge r.sender\neq m.sender\right\} \end{array}$$

as all the messages occurring within the time window of [*t*_1_, *t*_2_] seconds after *m* and sent by the team members other than *m*’s sender *m*.*sender*. We select the values for *t*_1_ and *t*_2_ based on how well they work for the purpose of binary classification of the messages of several manually annotated chat logs into true responses and non-responses, maximizing the classification result’s *F*_2_-score, with *F*_*β*_’s being defined in terms of the classification precision and recall as

$$\begin{array}{c} F_{\beta }=\left(1+\beta^{2}\right)\cdot \frac{\text{precision}\cdot \text{recall}}{\beta^{2}\cdot \text{precision}+\text{recall}}. \end{array}$$

**Fig 3:**
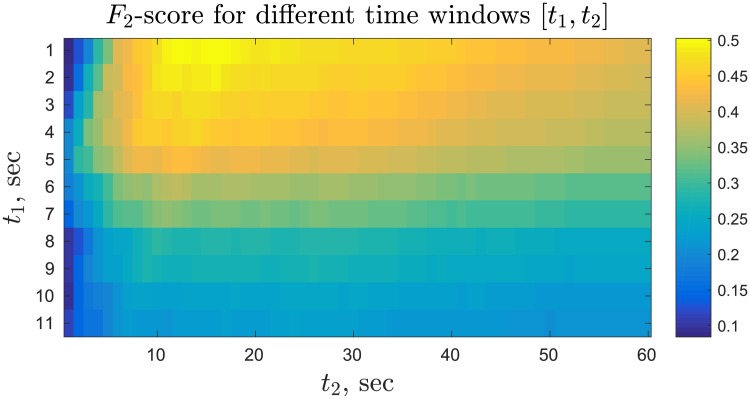
*F*_2_-scores for binary classification of log messages into responses and non-responses for different time windows [*t*_1_, *t*_2_] *sec*.

Based on the *F*_2_-scores for different combination of *t*_1_ and *t*_2_, shown in Fig 3, we choose the best response time window to be [*t*_1_, *t*_2_] = [1, 17] with *F*_2_ = 0.49.

As soon as we have defined a subset *R*(*m*) of responses for each message *m*, we define the *collaboration network W* whose nodes correspond to team members, and the weight *w*_*ij*_ of edge (*i*, *j*) is defined as

$$w_{ij}=\underset{\begin{array}{c} p.sender=i\\ q.sender=j\\ q\in R\left(p\right) \end{array}}{\sum }e^{-\rho \left|p.time-q.time\right|},$$ (2)

where the summation is performed over all suitable pairs (*p*, *q*) of messages *p* and *q* contained in the team’s chat log, and *ρ*—equal 0.15 in our experiments—is a constant parameter regulating how fast the likelihood of a message’s being a response degrades with the increase of the time gap between the two messages. The edges corresponding to team members who have not communicated with each other are absent from network *W*, although, many such networks appear to be dense. In addition to definition (2), we have used a simpler edge weight definition—with the exponential term’s being replaced with 1, thereby, capturing only the number of times person *j* responds to person *i* over the entire conversation—yet, such simpler definition resulted in the predictive models of worse quality.

**Fig 4:**
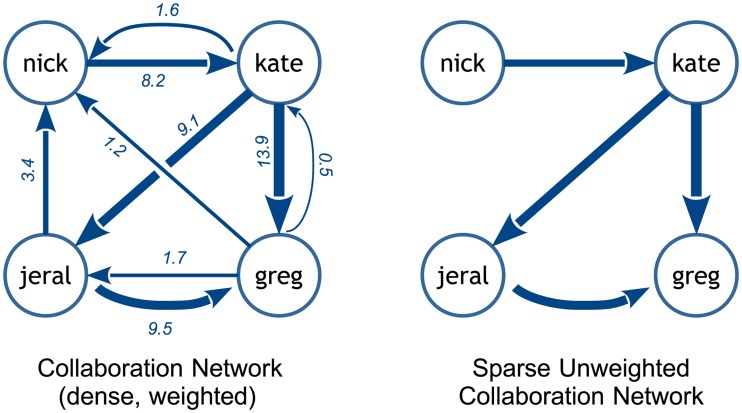
An examples of two types of collaboration networks constructed from a team’s chat log. In the shown sparse network *S*_50_, 50% of the lowest-weight edges of *W* have been dropped.

Having analyzed the text chat log for each team, we end up with a directed weighted network *W* reflecting who responds to whom while the team works on the tasks, as well as the amount and timing of these communication acts. In addition, for each network *W*, we define three *sparse unweighted collaboration networks S*_25_, *S*_50_, *and S*_75_, obtained from *W* by removing, respectively, 25%, 50%, and 75% of the lowest-weight edges and, subsequently, dropping all edge weights. Fig 4 provides an example of a dense weighted and a sparse unweighted collaboration networks derived from the partial chat log shown in Table 1.

For each team, we analyze both types of collaboration networks and extract from them the following network features, aggregated over all the nodes or edges of each network:

⊳ the mean and the standard deviation of node in- and out-degrees;
⊳ the difference of the mean and the standard deviation of in- and out-degrees; and
⊳ the mean edge reciprocity [38].

In addition to the above mentioned degree-based network features, we will discuss the related spectral features of our collaboration networks in Sec. 4.3.

The following network features are extracted only from sparse unweighted collaboration networks *S*_25_, *S*_50_, and *S*_75_:

⊳ edge density, defined as the ratio of the number of present edges to the maximum possible number of edges;
⊳ the network’s diameter;
⊳ the mean length of a shortest path;
⊳ the number of (weakly) connected components;
⊳ the number of strongly connected components;
⊳ the mean betweenness centrality of a node; and
⊳ the mean clustering coefficient of a node.

In addition to the network features, we extract from the chat logs the following team features, among whom the ones marked with † have been investigated in previous works [28, 29] for their potential connection with team performance.

† the total number of characters and words written by a team;
† the mean and the standard deviation of the number of characters and words written by a team member over all the members of a single team;
† the number of turns taken in the chat conversation;
⊳ the mean, the median, and the standard deviation of a delay between adjacent chat messages; and
⊳ the mean and the standard deviation of the quantified sentiment of a text message.

For the sentiment-related features on the list above, the sentiment of each text message was quantified as a real number from −1 (extremely negative) to 0 (neutral) to 1 (extremely positive). The sentiment quantification was performed using AlchemyAPI sentiment classifier, whose expected classification accuracy evaluated using a discrete set {−1, 0, +1} of sentiment values and the dataset of short text messages from www.sentiment140.com is 70%.

## 4 Results

In this section, we report our team performance prediction results. We use the general method of Sec. 3.1 together with, first, the baseline team features of Sec 2 and, then, the performance dynamics-based and the collaboration pattern-based features of Sec 3.2 and Sec 3.3, respectively.

### 4.1 Team performance via baseline team features

**Fig 5:**
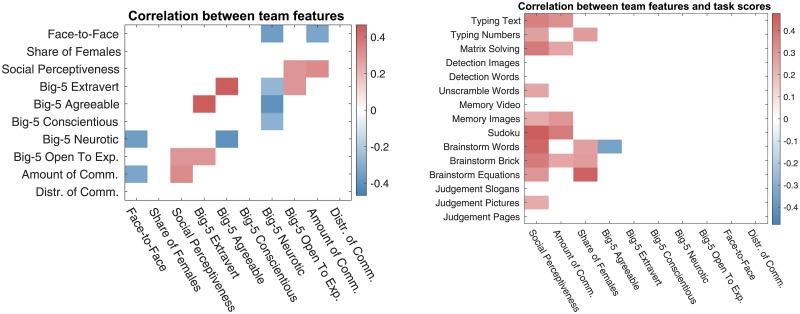
Correlation between baseline team features as well as their correlation with the task scores. Only statistically significant correlation values are displayed, with p-value threshold’s being 0.05. The p-values have been corrected using Benjamini-Hochberg procedure.

First, we analyze the correlation between the baseline team features as well as their correlation with the task scores. The correlation values are shown in Fig 5.

Among the baseline features, three features, namely social perceptiveness (*ρ* ∈ [0.24, 0.48]), amount of communication (*ρ* ∈ [0.26, 0.38]), and the share of females (*ρ* ∈ [0.27, 0.45]) are significantly correlated with team performance. These results replicate the original analysis of the same dataset as well as other previous work on collective intelligence [28, 29].

**Fig 6:**
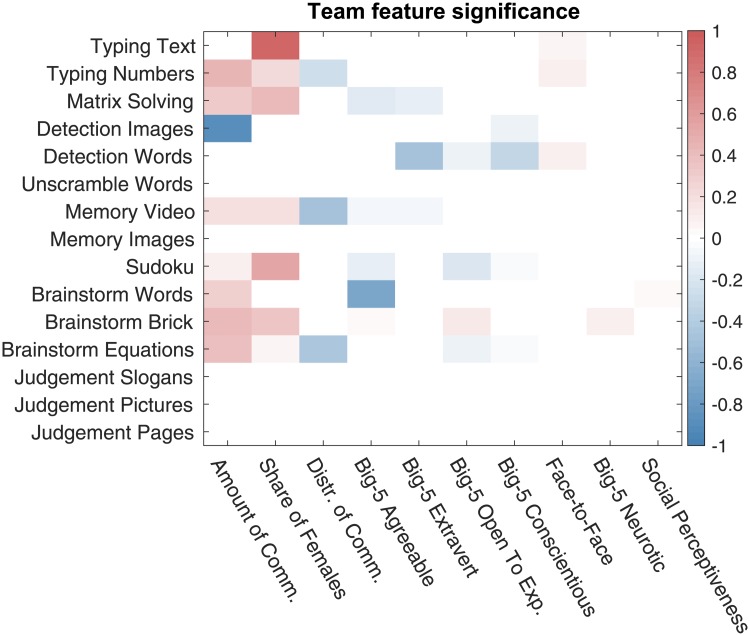
The team features emphasized in the regression models built with baseline team features. Each row includes the team feature coefficients of the best elastic net regression model for a given task. In the best models, the regularization is dominated by LASSO, with *λ*_1_: *λ*_2_ = 75%: 25% in the loss function (1).

Next, we use the baseline features for team performance regression modeling, as described in Sec. 3.1. The best models for each task are shown in Fig 6.

We see that the amount of communication as well as the proportion of females have a noticeable positive connection with team performance, while the distribution of communication (the standard deviation of the numbers of words written of spoken by team members) has a negative connection. Again, these results echo previous analyses of this and other similar datasets [28, 29]. We find two results that differ from previous analyses, however. First, *Social Perceptiveness* is *not* emphasized in the regression models, while—based on the correlation analysis as well as the results reported in the existing literature [28, 29]—we would expect it to have a positive connection with the teams’ performance on many tasks. This discrepancy may take place because the proportion of females and the social perceptiveness are strongly correlated, yet, in the best regression models, the effect of *ℓ*_2_-regularization is insufficient to group these two features equalizing their coefficients in the model, so the proportion of females feature likely “cloaks” the effect of the social perceptiveness feature. Second, unlike in previous analyses, we find that agreeableness and extraversion have a negative relationship with team performance. Perhaps, for example, groups that are very agreeable avoid conflict and perform worse, and perhaps those that are very extraverted spend more time on socializing instead of task performance.

**Fig 7:**
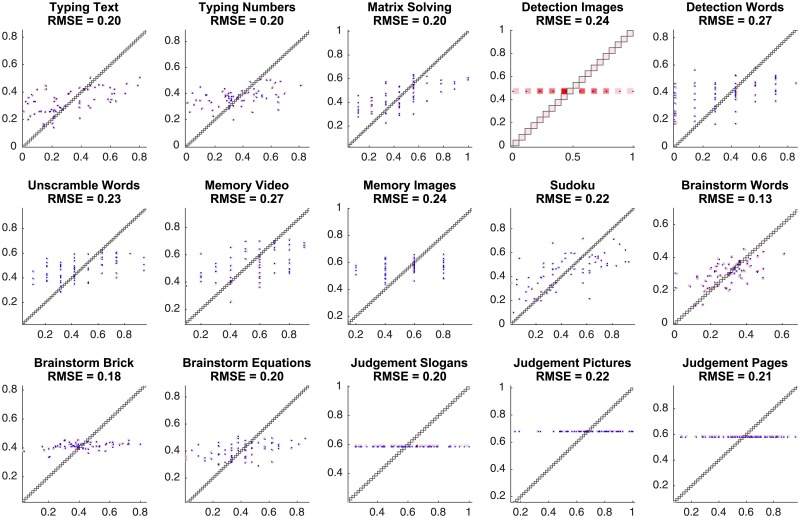
The prediction accuracy of the baseline best regression models for each task. On the displayed scatter plots, the x-coordinate corresponds to the true task score of a team, the y-coordinate corresponds to the predicted task score, and each point corresponds to a task score prediction for a single team. The root mean square errors (RMSEs) for predictions are also reported.

The prediction accuracy of the best regression model for each task is shown in Fig 7. We observe that for most of the tasks—excluding image detection as well as all three judgement tasks—we have found non-degenerate models for team performance. The models resulting in the lowest RMSE for the four excluded tasks are degenerate in that all their feature coefficients are approximately zero, and the score predictions are approximately equal to the mean scores for the respective tasks. We will use the obtained RMSEs later, to compare the predictive power of the baseline models with alternatives.

### 4.2 Team performance via historical performance dynamics

In this section, we analyze team performance using the general method of Sec. 3.1 and the team features derived from the dynamics of the teams’ performance on earlier tasks, as described in Sec. 3.2. More specifically, we perform the explanatory analysis of team performance with respect to the chosen features, as well as a quantitative analysis of the predictive quality of our model as compared to baselines. Later, in Sec. 5, we will show how to use the predictive model analyzed in this section for the purposes of optimal dynamic planning of workload in organizations.

**Fig 8:**
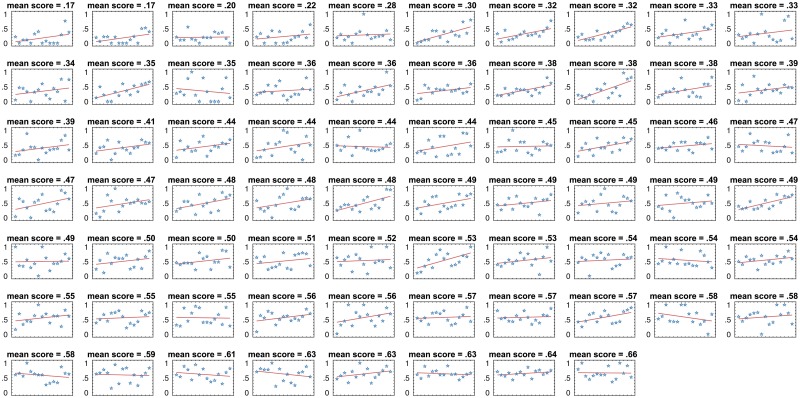
The series of scores for each of 15 tasks for each of 68 teams. The teams are arranged from the lowest- (top left) to the highest- (bottom right) performing with respect to the mean task score. Each score series is accompanied by the best fit line.

Prior to proceeding to the formal analysis, we would like to develop an intuition for how the historical dynamics of team performance is connected to the future team performance. To that end, in Fig 8, we display the task score series for all 68 teams, having ordered the teams based on their average performance, from the lowest- to the highest-performing team.

In Fig 8, it is easy to observe that

⊳ the higher-performing teams usually perform well as of the first task, while the lower-performing teams perform poorly on the first few tasks; and
⊳ the higher-performing teams perform well consistently, while the performance of the lower-performing teams noticeably changes through time.

**Fig 9:**
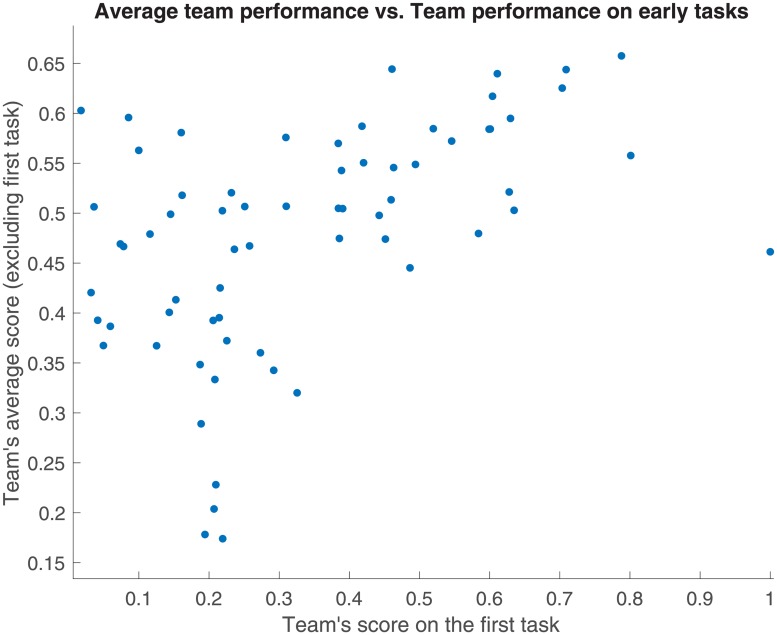
For each team, its average score over all but the first tasks (to avoid overfitting) is compared to its score on the first task. The two measures are significantly correlated (*ρ* = 0.50, *p* = 1.73 ⋅ 10^−5^).

The above observations become even more pronounced when we compare the teams’ average performance with their performance on early tasks as shown in Fig 9.

In order to formalize and quantify the above observations, we proceed with the correlation and regression analysis of team performance. First, we look at the correlation between the performance dynamics-based features and baseline team features.

**Fig 10:**
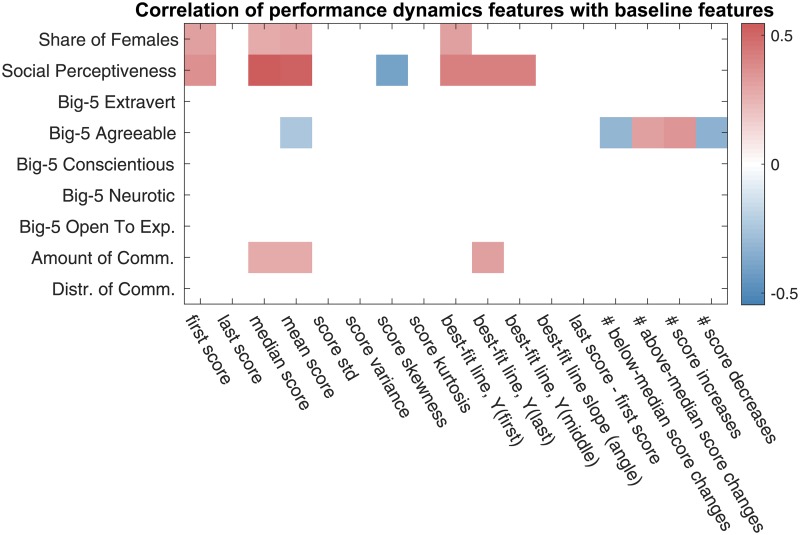
Correlation between the performance dynamics-based features of Sec. 3.2 and the baseline team features. Only the statistically significant correlation values are displayed, with the p-value threshold of 0.05. The p-values have been corrected using Benjamini-Hochberg procedure.

**Fig 11:**
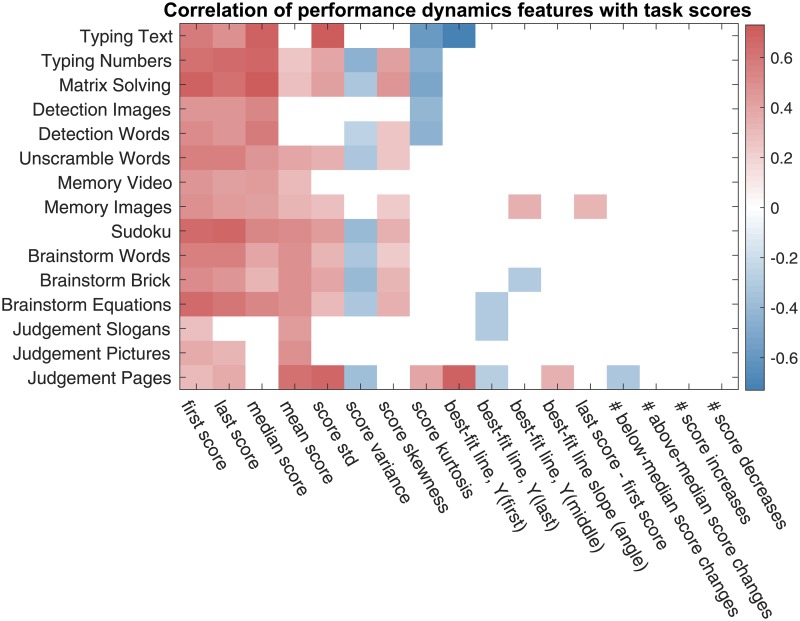
Correlation between the performance dynamics-based team features and task scores. Only the statistically significant correlation values are displayed with the p-value threshold of 0.05. The p-values have been corrected using Benjamini-Hochberg procedure.

Based on the correlation results shown in Figs 10 and 11, we make the following observations:

⊳ The proportion of females and the social perceptiveness—the features positively connected with team performance, as pointed out in Sec. 4.1—are positively correlated with the first, mean, and median task scores, as well as the corresponding features of the best fit line; the correlation values for the proportion of females are between 0.29 and 0.32, and the correlation values for the social perceptiveness are between 0.36 and 0.55.
⊳ The task scores are generally negatively correlated with the skewness (*ρ* ∈ [−0.45, −0.24]), and kurtosis (*ρ* ∈ [−0.58, −0.42], except *ρ*_*JudgementPages*_ = 0.40) of the score series; the latter quantify the dispersion of scores about their mean. This finding supports the earlier stated hypothesis about the performance consistency of the high-performing teams.
⊳ The Big-5 personality trait score characterizing agreeableness is slightly negatively correlated with the mean score (*ρ* = −0.25), yet, is positively correlated with the number of times a task score increases (*ρ* = 0.35) or, more generally, changes above the median score value (*ρ* = 0.32). These two findings are self-consistent, since, from what we have established above, the latter score dynamics is characteristic of lower-performing teams.

**Fig 12:**
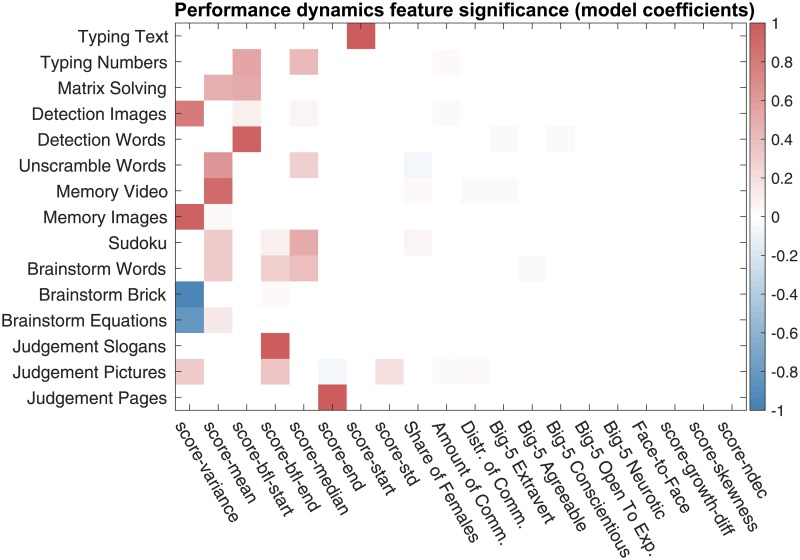
The features based on the dynamics of historical team performance emphasized in the best regression models.

**Fig 13:**
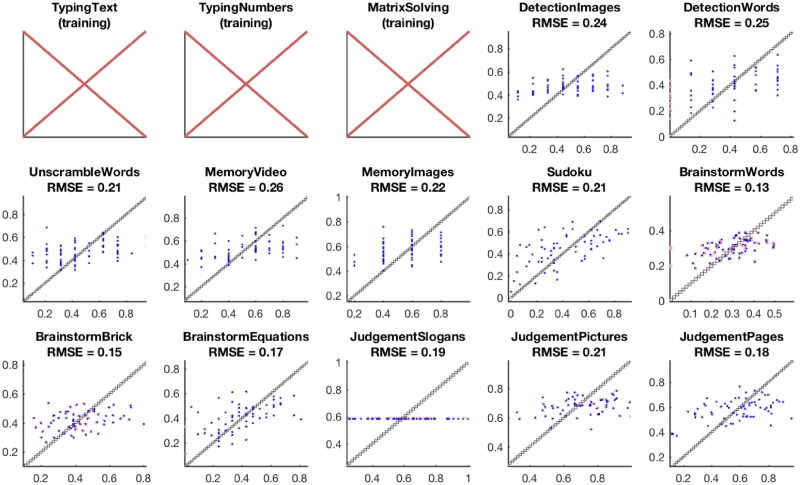
The prediction accuracy of the best regression models obtained using the team performance dynamics features. On the displayed scatter plots, the x-coordinate corresponds to the true task score of a team, the y-coordinate corresponds to the predicted task score, and each point corresponds to a task score prediction for a single team. Since prediction requires information about the teams’ performance on the previous tasks, the performance for the first three tasks is not predicted.

Next, we use the performance dynamics-based features in the regression analysis, executed as described in Sec. 3.1 with the following modification. When predicting the teams’ performance on a given task, we compute the performance dynamics feature values using only the preceding task, the teams’ performance on which has already been observed. Since our method relies on historical performance data, we predict team performance starting from the fourth task and further. The best regression models as well as their quality are shown in Figs 12 and 13, respectively.

Further, we will analyze the predictive power of our model relying on the performance dynamics-based features. Besides comparing its prediction accuracy to that of the method of Sec. 2 relying on the baseline features, we add into comparison several time series extrapolation methods that build time series models for each team individually: *Mean Oracle* (a baseline whose prediction equals the mean task score computed over both observed and not-yet-observed task scores), *Observed Mean* (the predicted score is the mean over the so-far observed task scores), *Naive Forecast* (the prediction equals the score for the immediately preceding task), *Least Squares* (predictions are obtained from a linear model fitted to the series of observed scores using least squares), and *ARMA* (an autoregressive moving-average model [39], with its parameters estimated via maximum likelihood). The prediction RMSEs for all the methods are reported in Table 2.

**Table 2:** Comparison of RMSEs for team performance prediction on 15 tasks using the general method of Sec. 3.1 with baseline features (Baseline) and the performance dynamics-based features (Dynamic), as well as using several standard time series extrapolation methods.

Method	Tasks														
Baseline (Sec. 2)	**.20**	**.20**	**.20**	.24	.27	.23	.27	.24	**.22**	**.13**	.18	.20	.20	.22	.21
Dynamic (Sec. 3.2)	×	×	×	**.24**	**.25**	**.21**	**.26**	**.22**	**.21**	**.13**	**.15**	**.17**	.19	**.21**	**.18**
Mean Oracle	×	×	×	**.21**	**.25**	**.20**	**.25**	**.23**	**.20**	.20	**.17**	**.17**	**.23**	**.29**	**.24**
Observed Mean	×	×	×	.27	**.25**	.24	.30	.27	**.21**	.21	.18	**.17**	.27	.32	.25
Naive Forecast	×	×	×	.30	.30	.35	.35	.34	.29	.29	.20	.22	.33	.28	.33
Least Squares	×	×	×	.42	.46	.41	.44	.36	.34	.35	.24	.26	.38	.37	.30
ARMA	×	×	×	.26	.33	.29	.32	.30	.32	.25	.21	**.18**	.27	.32	**.24**

The RMSEs corresponding to either unavailable or degenerate models are displayed in dark cells. Within each of two method categories, for each task / column, the best (lowest) RMSEs (±0.01) are displayed in bold font.

### 4.3 Team performance via collaboration network features

**Fig 14:**
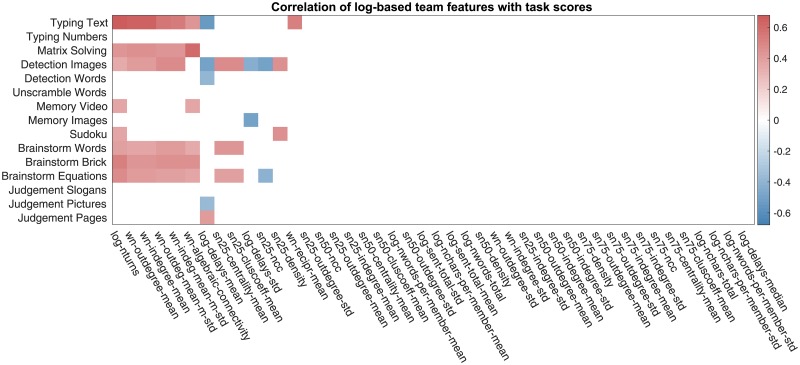
Correlation between the collaboration team features of Sec. 3.3 with task scores. Only the statistically significant correlation values are displayed with the p-value threshold of 0.05. The p-values have been corrected using Benjamini-Hochberg procedure. The features names of (dense weighted) collaboration networks have prefix “wn”, the feature names of the sparse unweighted collaboration networks with X% lowest-weight edges dropped are prefixed with “snX”, and the general chat log-based features have prefix “log”.

In this section, we will use the team features of Sec. 3.3 extracted from the text chat logs and, in particular, the team network representations for predicting team performance. We start with the correlation analysis, whose results are reported in Fig 14; and the correlation values for the top correlated features are reported in Table 3.

**Table 3:** Top log-based team features significantly correlated with task scores.

Team Feature	*ρ*, [min, max]	max **p-value**
# of turns in conversation	[-0.34,-0.68]	0.047
Node mean in- or out-degree	[-0.37,-0.65]	0.05
Node in- or out-degree, *mean* − *std*	[-0.38,-0.55]	0.025
Algebraic connectivity	[-0.35,-0.59]	0.04
Median delay between chat messages	[−0.53, −0.37] ∪ {0.41}	0.03

The correlation analysis in Table 3 suggests that there are two facets of communication noticeably connected to team performance.

⊳ Firstly, *the amount and*—due to the semantics of our collaboration networks—*frequency of communication are associated with higher team performance*. One fact supporting this statement is that the node degree-based features as well as algebraic connectivity, which have larger values in denser networks, both appear in Table 3 as noticeably correlated with team performance. Another fact is that the median delay between chat messages (as well as the number of turns taken in conversation—the feature investigated by Woolley et al. [29]) also show up as correlated. This finding confirms the results of Woolley et al. [29] and Engel et al. [28] regarding the amount of communication’s being positively related to team performance.

⊳ Secondly, and more prominently, *the well-connectedness of the collaboration network, and, more specifically, the uniformity of collaboration are associated with higher team performance*. Both Woolley et al. [29] and Engel et al. [28] found that the non-uniformity of communication—expressed through the *Communication Distribution* baseline feature (Sec. 2.2) measuring the variance in the amount of communication by team members—was negatively connected with the team’s collective intelligence. Our results in Table 3 allow us to extend and strengthen this conclusion, and apply it to team performance on individual tasks rather than an aggregate team performance measure, such as collective intelligence.

**Fig 15:**
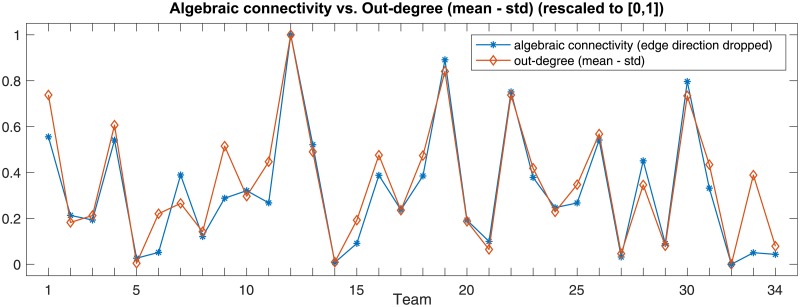
The relationship between the difference of the mean and the standard deviation (STD) of the out-degree of the collaboration network and its algebraic connectivity for each of 34 teams.

The differences of the mean and the standard deviation of the in- and out-degrees of the nodes of the weighted collaboration networks appear to be noticeably correlated with team performance. Intuitively, these features indicate how uniformly highly-connected the nodes of the network are—a large average node degree is indicative of high edge density, and a small standard deviation of the node degree indicates structural uniformity of the network. More formally, these node degree-based features are closely related to the algebraic connectivity of the network, that appears to be the network feature most correlated with team performance. Algebraic connectivity, defined as the smallest positive eigenvalue of the network’s Laplacian matrix [40], measures well-connectedness of the network through how robust it is to perturbations as well as how fast network processes, such as a random walk [41] or a consensus formation [42], unroll in it. Empirically, the rescaled out-degree-based feature and the algebraic connectivity of network closely follow each other, as shown in Fig 15.

It is clear, however, that one can deliberately design a team with only two members interacting a lot—making the average degree of their collaboration network high—yet, whose performance is low due to the lack of coordination between most of team members. To achieve high performance, the activity should be uniformly distributed among team members, as reflected in the robustness (algebraic connectivity) of their collaboration network. Because of that, it is very likely that in larger-scale sparse collaboration networks, the connection of degree summaries (mean, standard deviation) with team performance would wane, while the connection between the network’s robustness—expressed as the algebraic connectivity—and team performance would persist.

This latter result is particularly important, because there is no communication or collaboration uniformity measure in the existing literature that has been shown to be statistically significantly related to team performance on specific tasks.

We have also initially observed that the variance in the sentiment of conversation is negatively related to team performance, yet, this result turned out to be statistically insignificant after Benjamini-Hochberg correction.

**Fig 16:**
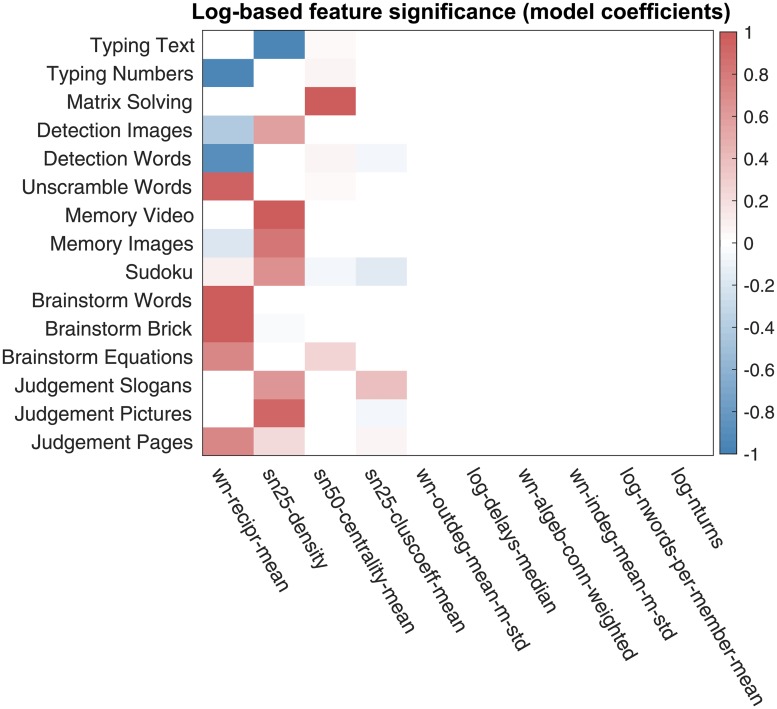
The chat log-based team features emphasized in the best regression models. Each row includes the team feature coefficients of the best elastic net regression model for a given task. The features names of (dense weighted) collaboration networks have prefix “wn”, the feature names of the sparse unweighted collaboration networks with X% lowest-weight edges dropped are prefixed with “snX”, and the general chat log-based features have prefix “log”.

Having performed the correlation analysis, we proceed with the regression analysis. For each task, we build a regression model, whose coefficients are shown in Fig 16. The features most emphasized in the best regression models are shown in Table 4. From the obtained results, we see that, similar to the above reported results of the correlation analysis, the features characterizing a network’s well-connectedness are emphasized in the regression models.

**Table 4:** Top 4 chat log-based team features emphasized in the best regression models.

Team Feature	min|*β*_*i*_|	max|*β*_*i*_|	mean|*β_i_*|
Mean edge reciprocity	0.02	0.99	0.66
Edge density, *S*_25_	0.03	0.99	0.63
Mean betweenness centrality, *S*_50_	0.01	0.99	0.18
Mean clustering coefficient, *S*_25_	0.01	0.38	0.07

The features are sorted by mean|*β*_*i*_|, where *β*_*i*_ is the model’s coefficient corresponding to the team feature.

**Fig 17:**
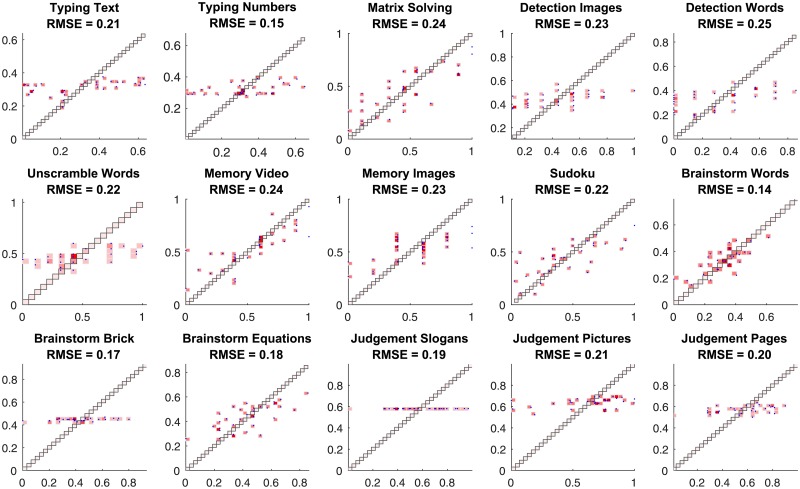
The accuracy of team performance prediction based on chat log-based and, particularly, network-based features. The best elastic net regression model is used for score prediction of each team on each task. On the displayed scatter plots, the x-coordinate corresponds to the true task score of a team, the y-coordinate corresponds to the predicted task score, and each point corresponds to a task score prediction for a single team. The root mean square error (RMSE) for predictions are reported.

The prediction quality of the best models for the team performance on each task is reported in Fig 17.

The quality of team performance prediction based on using collaboration features is compared to the prediction quality obtained using baseline and performance dynamics-based features, with the results shown in Table 5.

**Table 5:** Comparison of RMSEs for team performance prediction on 15 tasks using the general method on Sec. 3.1 with baseline features of Sec. 2 (Baseline), team performance dynamics-based features of Sec. 3.2 (Dynamic), and the features extracted from the text chat logs (Log-based), respectively.

Method	Tasks														
Baseline (Sec. 2)	**.20**	.20	**.20**	.24	.27	.23	.27	.24	**.22**	**.13**	.18	.20	.20	.22	.21
Dynamic (Sec. 3.2)	×	×	×	**.24**	**.25**	**.21**	.26	**.22**	**.21**	**.13**	**.15**	**.17**	.19	**.21**	**.18**
Log-based (Sec. 3.3)	**.21**	**.15**	.24	**.23**	**.25**	**.22**	**.24**	**.23**	**.22**	.14	**.17**	**.18**	.19	**.21**	.20
Mean Oracle	×	×	×	**.21**	**.25**	**.20**	**.25**	**.23**	**.20**	.20	**.17**	**.17**	**.23**	**.29**	**.24**
Observed Mean	×	×	×	.27	**.25**	.24	.30	.27	**.21**	.21	.18	**.17**	.27	.32	.25
Naive Forecast	×	×	×	.30	.30	.35	.35	.34	.29	.29	.20	.22	.33	.28	.33
Least Squares	×	×	×	.42	.46	.41	.44	.36	.34	.35	.24	.26	.38	.37	.30
ARMA	×	×	×	.26	.33	.29	.32	.30	.32	.25	.21	**.18**	.27	.32	**.24**

The RMSEs corresponding to either unavailable or degenerate models are displayed in dark cells. Within each of two method categories, for each task / column, the best (lowest) RMSEs (±0.01) are displayed in bold font.

From Table 5, we can conclude that on most of the tasks, the log-based performance predictions are more accurate than these of baseline predictions. Additionally, on six tasks, including the first three, the log-based predictions are more accurate than the ones based on the performance dynamics-based features. However, on most tasks excluding the first three, the performance dynamics-based predictions are superior to others.

## 5 Application

In order to demonstrate the usefulness of our team performance model of Sec. 3.2 and 4.2 relying on the features based on the dynamics of observed team performance, we will consider a practical application of this model to the problem of optimal online planning of workload distribution in an organization. Our goal here is to see how our predictive model can actually be used in practice as well as see how well it performs in comparison to alternatives.

Suppose that a manager of *n* teams needs to sequentially assign *m* tasks to the teams based on how well they are expected to perform on these tasks. Assuming that the work on each task is arbitrarily partitionable, let *ω*(*t*) ∈ [0, 1]^*n*^ ($1^{⊺}\omega \left(t\right)=1$) describe how the workload on task *t* ∈ {1, …, *m*} is distributed over all the teams, with *ω*_*i*_(*t*)’s standing for the share of work team *i* will perform on task *t*. If *s*(*t*) ∈ [0, 1]^*n*^ are the scores the teams will earn for task *t*’s completion, then the teams’ total score on task *t* is $s{\left(t\right)}^{⊺}\omega \left(t\right)$.

When tasks 1, …, (*t* − 1) have been completed, the manager is given the historical performance Hist^train^(*t*) of other teams who have already worked in the past on all the tasks up to *t* as well as the performance Hist(*t*) = 〈*s*(*k*) ∣ *k* = 1, …, *t* − 1〉 of the *n* currently managed teams observed so far. Given Hist^train^(*t*) and Hist(*t*), the manager’s goal is to predict the performance *s*^*est*^(*t*) of the teams on the next task *t*, and, based on this prediction, define workload distribution *ω*(*t*) on task *t*, so that

⊳ more work is assigned to the teams who—according to *s*^*est*^(*t*)—are expected to perform well on task *t*, yet,
⊳ the workload distribution is fair in that it does not happen that most of the work is assigned to a single team, expected to perform best on the given task, while the rest of the teams free-ride.

More formally, if the manager adopts workload distribution policy P, then the manager’s payoff R on task *t*, on which the teams’ actual scores are *s*(*t*), is defined as

$$\begin{array}{c} \text{R}\left(t,s\left(t\right),\omega \left(t\right)\right)=\underset{exploit}{\underset{︸}{s{\left(t\right)}^{⊺}\omega \left(t\right)}}-\underset{explore}{\underset{︸}{\alpha ∥\omega \left(t\right){∥}_{2}^{2}}},\\ \omega \left(t\right)=\text{P}\left(t,{\text{Hist}}^{\text{train}}\left(t\right),\text{Hist}\left(t\right)\right). \end{array}$$ (3)

In (3), the first summand describes the teams’ total score for task *t*’s completion, the second summand is the uniformity penalty ensuring fairness of workload distribution *ω*(*t*), and *α* ∈ [0, 1] is a constant parameter regulating the extent to which the fairness is emphasized as compared to the teams’ total score. Alternatively, the first and the second summands of (3) can be thought of as, respectively, emphasizing exploitation (of the knowledge of the teams performing best so far) and exploration (of the teams whose performance is yet largely unknown).

In what follows, we provide multiple workload distribution policies *P*—from the basic unaware of the past team performance to the more advanced based on our predictive team performance model—and compare their performance with respect to the manager’s cumulative payoff $\sum_{t=1}^{m}\text{R}(t,s(t))$ that the teams generate on a sequence of *m* tasks. More specifically, we consider the following workload distribution policies.

⊳ *Uniform Assignment*: It is a baseline workload distribution policy that assigns the same amount of work to each team, being ultimately fair

$$\begin{array}{c} {\text{P}}_{uniform}\left(t,{\text{Hist}}^{\text{train}}\left(t\right),\text{Hist}\left(t\right)\right)=1/n. \end{array}$$

⊳ *Mean Oracle*: This baseline workload distribution policy estimates the teams’ scores on task *t* as the average of the teams’ scores over *both* past and future tasks,

$$\begin{array}{c} s^{est}\left(t\right)=s^{est}=\sum_{i=1}^{m}s(i)/n, \end{array}$$

and the workload distribution *ω*(*t*) is chosen to be optimal with respect to the score estimates *s*^*est*^(*t*)

$${\text{P}}_{oracle}\left(t,{\text{Hist}}^{\text{train}}\left(t\right),\text{Hist}\left(t\right)\right)=\underset{\begin{array}{c} \omega \in {\left[0,1\right]}^{n}\\ 1^{⊺}\omega =1 \end{array}}{\text{arg}\;\text{max}}\underset{\text{R}\left(t,s^{est}\left(t\right),\omega \right)}{\underset{︸}{\left[s^{est}{\left(t\right)}^{⊺}\omega -\alpha ∥\omega {∥}_{2}^{2}\right]}}.$$ (4)

All the subsequent policies will use the same optimization procedure (4) for choosing *ω*, and will differ only in how they estimate the team scores *s*^*est*^(*t*).

⊳ *Naive Forecast*: Policy P_*naive*_ assumes that the teams’ scores on task *t* will be identical to their scores on task (*t* − 1), that is, *s*^*est*^(*t*) = *s*(*t* − 1).

⊳ *Observed Mean*: Policy P_*mean*_ defines the teams’ scores on task *t* as the means of the scores the teams received in the past, that is, $s^{est}\left(t\right)=\sum_{i=1}^{t-1}s(i)/n$.

⊳ *Least Squares*: Policy P_*LS*_ finds the best-fit—in the least squares sense—linear model for each team’s observed score series and uses it to estimate $s_{i}^{est}\left(t\right)$.

⊳ *ARMA*: Policy P_*ARMA*_ estimates $s_{i}^{est}\left(t\right)$ from an ARMA model, whose parameters are estimated using maximum likelihood.

⊳ *Performance Dynamics-based Prediction*: Policy P_*PD*_ estimates team performance *s*^*est*^(*t*) using our model of Sec. 3.2 and 4.2 that relies on the observed team performance dynamics features.

⊳ *Performance Dynamics-based Prediction with Outlier Control*: The previously described policy P_*PD*_ is based upon our predictive model for team performance, which is superior to the predictive models used by other policies in terms of the mean prediction accuracy, measured as the root mean square error (RMSE). However, while keeping a low RMSE, our predictive model can have a small number of outlier predictions that considerably deviate from their respective true performance values. The latter is an issue, since the workload distribution optimization problem (4) is non-robust to such outliers. Thus, we also consider policy P_*PDOC*_, which is a smoothened version of *P_PD_*, that does not allow its score predictions ${\hat{s}}^{est}\left(t\right)$ to deviate by more than *ϵ* = 0.3 fraction from their so-far observed mean values

$$\begin{array}{cc} mean_{k} & =\sum_{i=1}^{t-1}s_{k}\left(i\right)/n,\\ {\hat{s}}_{k}^{est}\left(t\right) & =\max\{\left(1-\epsilon \right)mean_{k},\min\left\{\left(1+\epsilon \right)mean_{k},s_{k}^{est}\left(t\right)\right\}\}, \end{array}$$

where scores estimates $s_{k}^{est}\left(t\right)$ are obtained as in policy P_*pd*_.

While most of the policies on the list above base their score predictions solely on the observed performance Hist(*t*) of the currently managed teams, the two last policies that use our team performance prediction model are the only ones also exploiting the “training data” Hist^train^(*t*) that incorporates the historical performance of previously managed teams. Thus, the main goal of the comparison of the workload distribution policies is to understand whether and to what extent availability of this training data helps maximize the organization’s performance relatively to the policies that rely solely on the observed past performance of the teams working on tasks.

We evaluate the above described workload distribution policies using our data about the performance of 68 teams on 15 tasks. We assume that 51 teams (75%), having already worked on similar tasks, comprise the training data Hist^train^, and we sequentially perform workload distribution for each task among the remaining 17 teams. To ensure robustness of the obtained experimental results, the experiments are repeated over 100 random 75%-25% splits of teams, and the mean results are reported. We use *α* = 0.2 in the definition of the payoff $\text{R}\left(t,s\left(t\right),\omega \left(t\right)\right)=s{\left(t\right)}^{⊺}\omega \left(t\right)-\alpha {∥\omega \left(t\right)∥}_{2}^{2}$, making sure that the payoff mostly depends on assigning more work to the teams expected to perform well and, to a lesser extent, on the workload distribution uniformity.

**Fig 18:**
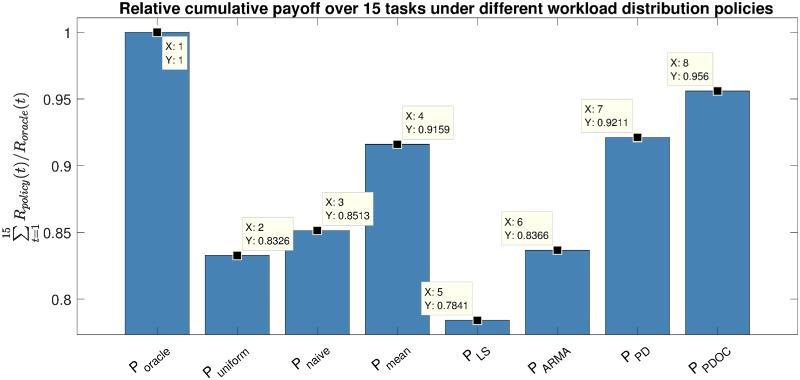
Relative cumulative payoffs over a sequence of 15 tasks corresponding to different workload distribution policies. The payoffs are scaled by the payoff of the oracle baseline policy.

**Fig 19:**
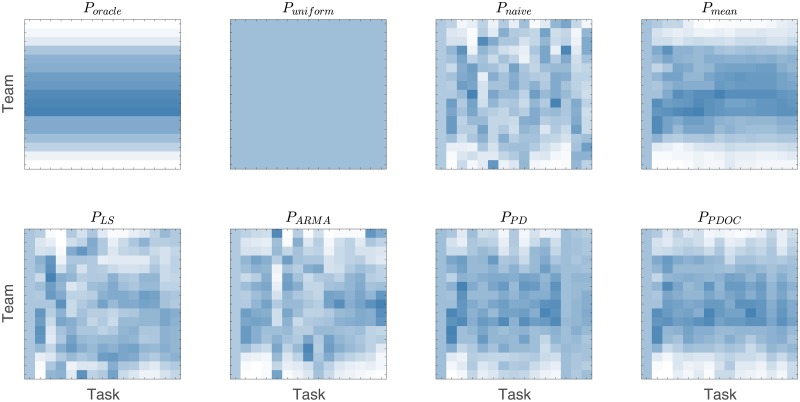
Workload distributions *ω*(*t*) chosen by each policy on each of 15 tasks. The workload distributions of first two baseline policies are time-invariant. Other policies, relying on the use of historical data, use *P*_*mean*_ if not enough historical data is available (for example, both least squares and ARMA fitting require at least 2 observed data points). For the first task, *P*_*mean*_ chooses a uniform workload distribution, as in *P*_*uniform*_.

The cumulative payoffs obtained using each of the workload distribution policies scaled by the cumulative payoff of the oracle baseline policy P_*oracle*_ are shown in Fig 18. The average optimal workload distributions for each policy are shown in Fig 19.

We can see that policy *P*_*PDOC*_ that relies on our predictive model for team performance results in the highest cumulative payoff, producing rather uniform workload distributions. However, it is also important to notice that the outlier control in *P*_*PDOC*_ is essential for the policy to noticeably outperform a much simpler intuitive policy *P*_*mean*_.

## 6 Discussion

In this work, we have focused on the analysis of the dynamics of the team process for the purposes of understanding and predicting team performance. We have introduced the team performance predictive models based on the properties of the historical team performance dynamics, as well as on the explicitly captured collaboration patterns occurring in teams during task completion. We have also shown how our performance dynamics-based predictive model can be used in an application.

**Dynamics of Historical Team Performance Predicts Future Performance:** Our first qualitative finding is that the dynamics of team performance on early tasks in a sequence is predictive of team’s performance on future tasks. More specifically, high-performing teams start doing so early, while low-performers start poorly, yet, in many cases, consistently improve their performance over time.

One possible explanation for this observation is that, even though all the tasks in a sequence were simple enough so that every team member was able to tackle them, some teams might have nevertheless been worse prepared for task solving using the online web-based interface. However, this explanation is challenged by the fact that every participant of the study, prior to approaching the tasks the performance on which is evaluated, had practiced on non-scored tasks.

**Well-Performing Teams Have Well-Connected Collaboration Networks:** Our second qualitative finding is that there is a strong connection between the explicitly captured collaboration patterns in teams and these teams’ performance. We find that better performing teams have collaboration networks which are better connected, as manifested by their algebraic connectivity, have a more uniform connectivity structure, as manifested by the node degree-based features as well as edge reciprocity, and are denser. The network density’s connection to team performance is expected, as it has been reported in existing literature that better-performing teams communicate more [28]. If we assume that the amount of communication is commensurate with the degree of team members’ cooperativeness, this result is not surprising, as cooperation is expected to facilitate better performance [13].

On the other hand, the positive relationship between the amount of collaboration and performance is contrary to some existing works that focus on either usefulness or the cost of intra-team communication. As one example, Kanawattanachai and Yoo [43] report that, in the context of studying transactive memory systems, communication becomes less important with time, when the group’s shared knowledge of each other—affecting efficient expertise location inside the team—reaches a certain saturation point. In the study where our data is obtained from, however, the team members were strangers, and operated on a much shorter time scale (1 hour), which left not enough time for teams to reach the point where collaboration starts having diminishing returns. As another example, Hansen [44] argues that the need for maintenance of redundant links in knowledge networks can adversely impact performance outcomes when the knowledge being tranferred throught the network is codified. In our case, however, all the knowledge transferred during task completion is non-codified, and, according to Hansen, redundant links in such an environment should actually facilitate performance. As the final example, Hollingshead [45] reports that the teams with less of a need to explicitly coordinate—such as those in close relationship—perform better when they are not communicating, whereas communication has a positive effect on performance for stranger teams. Teams in our data composed of individuals who were not known to each other, and, thus, likely benefited from high volume of communication.

While the positive connection between the amount of collaboration and the resulting team performance is not so surprising, it is less obvious, however, why the well-connectedness and structural uniformity of (a potentially sparse) network is connected to high performance, as the tasks themselves do not impose any communication or collaboration uniformity requirements upon teams. One possible explanation is that a network’s well-connectedness characterizes how fast a consensus can be reached in such a network, and the latter may contribute to the team’s better outcomes. Alternatively, the structural uniformity of a collaboration network may help efficiently establish “joint intentions” [46], which formalize team members’ commitments and responsibilities and, in turn, facilitate effective teamwork. Our formal analysis of collaboration and its connection to team performance complements several related results in existing literature. Losada [47] found that the connectivity of a team—as measured by the cross-correlation of time series of “speech acts” of team members—is predictive of the team’s performance. Stewart [12] mentions in his review of team performance factors that “high coordination improves intra-team processes by opening communication channels, building feelings of esprit de corps, and reducing social loafing.” Balkundi and Harrison [48] state that “teams with densely configured interpersonal ties attain their goals better”. Finally, Engel et al. [28] find that the standard deviation of the amount of communication by team members in the process of task completion is positively connected to the team’s collective intelligence factor. In our work, we formalize and reinforce the claim about the positive effect of team “well-connectedness” upon team performance through the precise analysis of collaboration networks and their metrics. Overall, our study of network-based models supports a conclusion that *in order to form a well-performing team, besides selecting socially apt individuals, one should create a work environment that would promote team members’ efficiently communicating in a reciprocated fashion, making sure that the structure of the communication network facilitates efficient spread of processes—such as consensus formation—in this network*.

**Quality of the Predictive Models:** Finally, our third finding is that relying on such team features as those characterizing the team’s performance dynamics on early tasks and those explicitly quantifying the process of collaboration happening inside teams through network-level features results in higher-accuracy predictive models, with the prediction errors varying between 15% and 25% (RMSEs varying between 0.15 and 0.25).

**Applications:** Additionally, we have studied our model relying on the dynamics of the observed team performance in a practical application of optimal online planning of workload in an organization. We have observed that the online workload distribution policy based on our model augmented with an outlier control mechanism results in the highest cumulative payoff for the organization, as compared to the policies obtaining predictions through extrapolating the score series for each team individually.

**Limitations:** One limitation of our study, coming together with the primary data [28], is the sample size—68 teams / 272 persons—which may be considered fairly small, especially, if compared to the analyses performed at the level of an individual, rather than a team. However, it is important to note that research on teams often involves greater challenges in data collection than research on individuals, due to the impact of individual partipants’ no-shows and dropout on the entire team-level data. In addition, the data we used is adequate for the current study for two reasons. First, despite the sample size being not large, findings from the primary data replicated previous research on collective intelligence [29] and was replicated by subsequent research on the same topic [49]. This suggests that stability and replicability of study findings were not compromised by sample size. In addition, the data is well suited for answering our main research question on dynamics of collaboration. Examining the latter requires data on teams’ performance on multiple sequential tasks. The data used in this study provides a sufficiently large number of tasks that were administered in a standardized fashion. Other similar studies on collective intelligence, such as [50] and [51], include much fewer (3-5) tasks, thus, making it hard to model collaboration dynamics reliably.

**Future Work:** There are a few questions that our study leaves open. First, we have yet to establish the causal relationship between the collaboration dynamics and team performance. More specifically, it is important to understand whether incentivizing particular collaboration patterns would actually result in a higher team performance, or these patterns are just “symptoms” of high performance caused by some other factors, such as team members’ compatibility in psychological traits or social aptitude. Answering this question is an important direction for future research.

In addition, how generalizable our findings are beyond the laboratory deserves further attention. One may argue that the tasks in our data are fairly simple compared to the type of tasks workers encounter in the workplace, and question whether our models will be able to account for dynamics in the “real” teams in organizations. The tasks used in this study [29] were not designed to exactly mirror the tasks encountered in the workplace, but rather to capture an underlying factor that leads teams to perform well. Thus, the tasks in the test battery are different from the real-world tasks in the same way the tasks from the IQ test are different from the real problems people tackle daily. Nonetheless, we can expect that the group performance on a diverse group of “simple tasks” would transfer to that on other tasks, since, as in the case with the IQ, “…the concept of measurable human intelligence is based on a fact that …[p]eople who do well on one mental task tend to do well on most others, despite large variations in the tests’ contents and methods of administration” [29]. Indeed, the research on collective intelligence found that collective intelligence measured using simple cognitive tasks predicted groups’ later performance on more complicated tasks including building structures following strict building codes [29] and playing online multiplayer games [49], which require multi-faceted, fast coordination among team members. Future research should examine whether the findings of this study would still apply to teams that work on complex tasks over an extended period of time.

Finally, we encourage future research to consider an even wider range of tasks, including tasks with high interdependence which would require high degree of coordination. The tasks used in our experiments—described in S1 Text—do not have “hard” collaboration requirements, unlike, for example, the CRONUS tasks of [18]. We observed in our data that, despite no formal collaboration requirements, self-organization happens among team members in varying degrees. And the effect of collaboration varies across tasks. Fig 16 shows that density of the collaboration networks is positively connected with team performance on memorizing, detecting, and judgment tasks, yet, is negatively related to team performance on the text typing task. The situation is similar with the mean edge reciprocity measure and the number typing task. These observations seem to suggest that the amount of communication among team members may not have substantial impact on simple execution tasks as long as team members efficiently split the work and execute their respective assigned work chunks. Execution tasks do require coordination, but perhaps at a more implicit level. Memorizing, detecting, and judgment tasks, on the other hand, can greatly benefit from actively pooling together memories, points of view, and expertise of multiple performers. Overall, the tasks used in the primary data are fairly simple. However, our findings suggest that collaboration dynamics and network structures are important factors that contribute to strong team performance, even on fairly simple tasks. With more complex, and highly interdepedent tasks, we think that understanding of fine-grained dynamics of team collaboration will be even more important.

## Supporting information

S1 Dataset Dataset and description of the team features, task scores, and communication logs used in analyses; correlation- and regression-related values obtained in the experiments. (ZIP)

S1 Text Descriptions of the tasks. (PDF)
