# Supplementary material for: Dynamics of collective performance in collaboration networks
Source: PLoS One. 2018 Oct 10;13(10):e0204547. doi: 10.1371/journal.pone.0204547 (PMC6179230; doi:10.1371/journal.pone.0204547)
Supplement: S1 Text — (PDF) [file pone.0204547.s002.pdf]

S1 Text

Descriptions of the tasks

▷ Executing

TypingText, TypingNumbers: typing a complex text or a series of numbers in a shared online document, being rewarded for correctly typed text or numbers and penalized for errors.

▷ Sensing

DetectionWords, DetectionImages: judging object frequency-related characteristics, such as the most or least frequent object, of a large grid of words or images.

▷ Generating

BrainstormWords: generating as many as possible words starting with "S" and ending in "N".

BrainstormBrick: generating as many as possible ideas on how to use a brick.

BrainstormEquations: generating as many as possible arithmetic expressions equal 10 using operators $+$, $-$, $\div$, $\times$, in which the digits between 2 and 8 occur only once per equation.

▷ Choosing

MatrixSolving: collectively completing an 18-item subset of Raven's Advanced Progressive Matrices test, that includes 3-by-3 numeric matrices, each having the lower-right corner empty. The goal is to fill that missing element in each matrix based on the values of the matrix' other elements.

UnscrambleWords: unscrambling as many as possible out of 24 randomly scrambled words, with the performance's being determined by the number of correctly recovered words.

Sudoku: collectively solving a Sudoku puzzle. A group's performance is based on the proportion of the correctly filled Sudoku grid values.

JudgementSlogans, JudgementPictures, JudgementPages: predicting how a larger population would judge a slogan, a picture, or a number of pages in a book. A group's performance is based on the proximity of the group's members' answers to the ground truth determined through an Amazon Mechanical Turk poll.

▷ Memorizing

MemoryVideo, MemoryImages: recollecting features of a video or an image.
